# Feasibility and Acceptability of Bright Light Therapy Glasses for Older Women

**DOI:** 10.1093/geroni/igaf122.1804

**Published:** 2025-12-31

**Authors:** Linlin Ding, Meina Zhang, Chelsea Howland, Chooza Moon

**Affiliations:** University of Iowa, Iowa City, Iowa, United States; UT Health, United States; University of Iowa, Iowa City, Iowa, United States; University of Iowa, Iowa City, Iowa, United States

## Abstract

Women’s later years bring notable shifts in mood, sleep, and cognitive abilities influenced by menopause. Bright light therapy (LT) may help regulating circadian rhythms, improving sleep patterns, as well as enhancing emotional and cognitive functions. This study aimed to evaluate the feasibility of using bright light therapy glasses, contributing to preventive strategies that promote women’s health. The intervention group participants were instructed to wear Re-Timer glasses (500 lux light at the eye) for at least 30 minutes each morning upon waking for two weeks. The control group participants received a sleep health education booklet. Participants completed an acceptability questionnaire and open-ended structured questions to receive feedback. A total of 9 participants (mean age (SD) = 61.78 (8.47)) were randomly assigned to either the intervention group (n = 4) or control group (n = 5) and successfully completed the protocol. The majority (77.78%, n = 7/9) expressed strong satisfaction with involvement in the study. Among those in the intervention group, 100% (n = 4/4) participants strongly agreed that the Re-Timer glasses were acceptable. Additionally, 75% (n = 3/4) reported using Re-Timer glasses daily with an average of 32.23 minutes per day (range: 30-60 minutes). Participants reported improvements in focus and feeling refreshed throughout the day. However, some noted eye fatigue, difficulty using the glasses in low-light conditions, and issues with fit. Overall, participants reported the study was feasible and acceptable. Additional well-structured randomize controlled trial with larger sample sizes are needed to further validate their effectiveness.

